# Rapid small intestinal permeability assay based on riboflavin and lactulose detected by bis-boronic acid appended benzyl viologens

**DOI:** 10.1016/j.cca.2014.09.031

**Published:** 2014-10-06

**Authors:** Angel Resendez, Md Abdul Halim, Caroline M. Landhage, Per M. Hellström, Bakthan Singaram, Dominic-Luc Webb

**Affiliations:** a Department of Chemistry and Biochemistry, University of California at Santa Cruz, Santa Cruz, CA 95064, United States; b Department of Medical Sciences, Gastroenterology and Hepatology Unit, Uppsala University, 751 85, Uppsala, Sweden

**Keywords:** Intestinal permeability, Organoborane, Gastroenterology, Lactulose, Mannitol, Riboflavin

## Abstract

**Background:**

Although organoboronic acids are efficient high-throughput sugar sensors, they have not been pursued for gut permeability studies. A modification of the lactulose/mannitol assay is described by which small intestinal permeability is assessed at the time of urine collection using a lactulose/riboflavin ratio.

**Methods:**

Volunteers ingested 50 mg riboflavin and either 5 g mannitol or 10 g lactulose. Urine was collected for 6 hrs. Riboflavin was assayed by autofluorescence. Riboflavin was removed by C18 solid phase extraction. Lactulose and mannitol were then assayed using 1,1′-bis(2-boronobenzyl)-4,4′-bipyridinium (4,4′oBBV) coupled to the fluorophore HPTS.

**Results:**

The temporal profile over 6 hrs for riboflavin paralleled mannitol. Riboflavin recovery in urine was 11.1 ± 1.9 % (mean ± SEM, n = 7), similar to mannitol. There was selective binding of 4,4′oBBV to lactulose, likely involving cooperativity between the fructose and galactose moieties. Lower limits of detection and quantification were 90 and 364 μM. The lactulose assay was insensitive to other permeability probes (e.g., sucrose, sucralose) while tolerating glucose or lactose. This assay can be adapted to automated systems. Stability of 4,4′oBBV exceeds 4 years.

**Conclusions:**

Riboflavin measured by autofluorescence combined with lactulose measured with 4,4′oBBV represents a useful new chemistry for rapid measurement of intestinal permeability with excellent stability, cost and throughput benefits.

## 1. Introduction

Intestinal permeability allows for restrictive leakage of molecules and ions below ∼0.4 nm (MW ∼250 Da) from the gut lumen into blood circulation. This “paracellular” leakage occurs through tight junctions between epithelial cells comprising the intestinal mucosa and varies between individuals. Other molecules pass through the microvilli of the epithelial cells by “transcellular” transport. Elevated paracellular leakage has been implicated in many human disorders with immunological components, including type 1 diabetes mellitus [1], obesity and type 2 diabetes mellitus [2], inflammatory bowel disease (i.e., Crohn's disease and ulcerative colitis), celiac disease and irritable bowel syndrome [3–5], Parkinson's disease [6], environmental enteropathy [7] and cancer [8]. There are comparable associations to laminitis in horses [9]. Relevant to human and veterinary medicine are drug induced lesions and gut toxicity, as for instance by chemotherapy [10] or NSAIDs [11]. Future researchers, drug developers and care givers working with diseases not traditionally within the gastroenterology domain will seek assistance from this discipline due to rising interest to quantify gut permeability.

Gastroenterology units have limited resources to assay large numbers of urine samples for gut permeability to meet the anticipated demand. The classical sugar absorption test for small intestinal permeability uses lactulose to quantify abnormal paracellular leakage [12]. This is referenced against mannitol, giving a lactulose/mannitol ratio. Although mannitol is absorbed by the paracellular route, a low mannitol value in this test is regarded to reflect nutrient deficiency through reduction in nutrient transporters. Neither lactulose nor mannitol has intrinsic absorbance or fluorescence permitting rapid, direct quantification. Typically, two different HPLC configurations (e.g., ion exchange and reverse phase) are coupled to expensive detectors, such as mass spectrometers [13]. Alternatively, NAD(P)H-coupled enzyme assays are used [14,15], but require considerable time and cost.

Recent years have seen breakthroughs in molecular biology and organic chemistry that should facilitate simpler, faster and less expensive assessment of small intestinal permeability. For instance, riboflavin has been found to be almost entirely absorbed through the type 2 riboflavin transporter (RFT2) at apical epithelial membranes in the small intestine [16]. Loss of RFT2 expression results in severe riboflavin deficiency [17]. Hence, appearance of riboflavin in urine during 6 hrs after oral ingestion reflects absorption at duodenum and jejunum. Riboflavin's intrinsic fluorescence suggests a methodological advantage over mannitol while reflecting absorption of an actual nutrient.

**Fig. 1:**
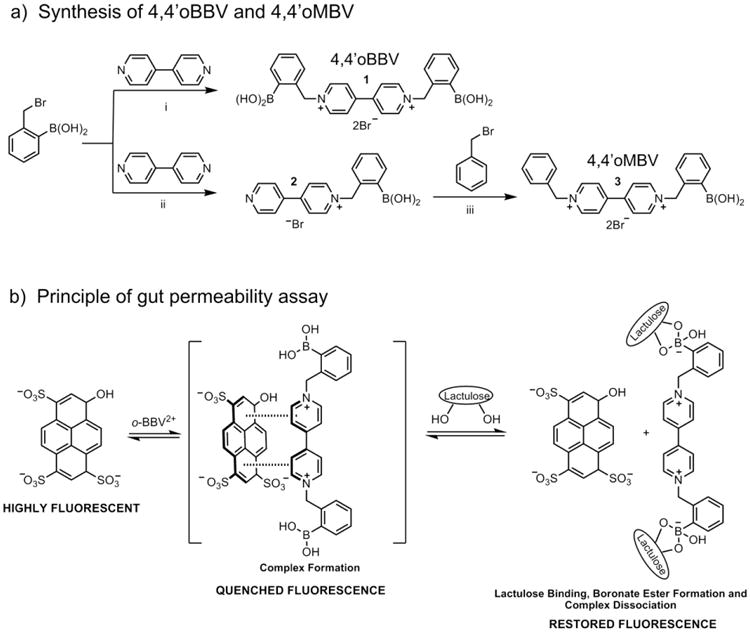
Synthesis of organoborane sugar sensors (a) and principle of fluorescence assay for urine lactulose or mannitol (b).

Much work has been done in using organoboronic acids to quantify sugars [18–20]. Focus has been on glucose related measurements, and these have had success in clinics; eight validated HbA1c assays employ organoboranes [21]. However, organoboronic acids are typically more sensitive to fructose and to lactulose, likely due to the fructose moiety. Although less sensitive, they can also bind mannitol. We reasoned that the intestinal permeability test could be simplified by 1) replacing mannitol with riboflavin and 2) removal of riboflavin by C18 solid phase extraction and measuring lactulose with an organoboronic acid coupled to a fluorophore. Benefits include rapid assessment of urine samples and negligible price. Reagents can be stored on ready to use plates in advance of measurement. We therefore synthesized the bis-boronic acid appended viologen 1,1′-bis(2-boronobenzyl)-4,4′-bipyridinium (compound **1**)(4,4′oBBV). For comparison, a mono-boronic analog (compound **3**)(4,4′oMBV) with increased water solubility was also synthesized (Fig. 1). Both can be coupled to fluorophores. These were first used to determine if riboflavin can replace mannitol and then to quantify lactulose.

## 2. Methods

### 2.1. Preparation of boronic acid viologens 1 (4,4′oBBV) and 3 (4,4′oMBV)

The reaction scheme with compound numbering is shown in Fig. 1a. Synthesis of 4,4′oBBV was as we reported earlier [22]. For 4,4′oMBV, 2-bromomethylphenyl boronic acid was reacted with excess 4,4′-bipyridyl in acetone to afford the mono-substituted 4,4′bipyridyl adduct (compound **2**). Combining excess compound **2** with benzyl bromide in a solvent mixture of MeCN and MeOH yielded 4,4′oMBV (compound **3**) after precipitation from the reaction mixture with acetone. Reagents and conditions were: (i) dimethylformamide, 55 °C, 48 hrs, 90% (compound **1**); (ii) acetone, 25 °C, 2 hrs, 70% (compound **2**); (iii) MeCN, MeOH, 55 °C, 24 hrs, 86% (compound **3**). Chemicals were from Sigma Aldrich (St Louis MO, USA) unless stated otherwise.

Fig. 1b illustrates the molecular mechanism behind the organoborane based fluorescent lactulose assay. The sensing ensemble is comprised of an anionic fluorophore, 8-hydroxypyrene-1,3,6-trisulfonic acid (HPTS) and a boronic acid-appended viologen (4,4′oBBV or 4,4′oMBV). HPTS forms a weak ground state complex with the cationic viologen sugar receptor, quenching its fluorescence. Ground state complex formation between the anionic fluorophore and cationic viologen sugar receptor facilitates an electron transfer from the fluorophore to the viologen, decreasing fluorescence. At pH ∼7.4, the cationic boronic acid viologen receptor has a high intrinsic affinity for cis-diols, which upon binding, partially neutralizes the charge of the viologen. This is caused by an equilibrium shift from the neutral boronic acid to the anionic boronate ester, lowering its affinity for HPTS, giving increased fluorescence.

### 2.2. Baseline absorbance spectra of urine

Because the assay uses spectroscopic methods, urine samples were evaluated for interfering absorbance. Urine was collected from volunteers, including subjects with suspected gut hyperpermeability. They were instructed to drink 0.5–1.0 L water the night before and in the morning ∼3 hrs prior to sample collection. The first morning urine was voided. No food or additional beverages were consumed until after the baseline sample was collected. This reflects current practice prior to initiating the permeability test. Absorbance was scanned from 380 to 700 nm at the time of collection. Below 380 nm, samples are essentially opaque. Absorbance is negligible from 700 to 1000 nm.

### 2.3. Temperature and solubility of reagents

A BioRad C1000 thermal cycler with CyberGreenfilters (Exc 470, Em 520 nm) was used to determine the thermal properties of the assay. A smaller set of samples were checked at 404/535 nm on the plate reader normally used for the permeability test (see below). Aliquots of 6 μL of a 4× premix containing 500 μM 4,4′oBBV and 16 μM HPTS in 100 mM sodium phosphate buffer at pH 7.4 were distributed onto PCR plates (HSP-9601, BioRad, Hercules, CA, USA). Then, 18 μL urine samples containing serially diluted 0 to 80 mM lactulose were distributed into the wells. Plates were sealed with clear plate tape and assayed from 5 to 70 °C.

### 2.4. Permeability test in humans

Subjects consumed 0.5–1.0 L water the night before and in the morning ∼3 hr prior to sample collection. The first morning urine was voided. No food or other beverages were consumed prior to the test. Permeability probes were ingested after baseline urine collection. Doses were 50 mg riboflavin (Freeda Vitamins Inc, Long Island City, NY, USA) and 5 g mannitol or 10 g lactulose (15 mL at 0.67 mg/mL; Meda AB, Solna Sweden). Test subjects were permitted to drink water or coffee as desired. Light snacks were permitted after the fourth hr. Urine volumes were recorded and 50 mL was retained for analysis. Studies were carried out in Sweden according to ethical approval Dnr 2010/184 held at Uppsala University, Sweden. In this jurisdiction, lactulose, mannitol and riboflavin are available over the counter.

### 2.5. Urine assays

#### 2.5.1. Sample collection

Volume of urine collected was recorded and 50 mL was first centrifuged 2500 RCF, 4 °C, 10 min. 100 μL supernatant was set aside for riboflavin analysis, the remaining supernatant was frozen at −20 °C for later mannitol or lactulose analyses.

#### 2.5.2. Riboflavin assay

The above 100 μL urine set aside and standards prepared in pooled baseline samples were diluted in 900 μL EtOH, vortexed and centrifuged. Supernatants were pipetted 40 μL/well in duplicate into plates (#3694, Corning, USA). Fluorescence (Exc/Em 450/580 nm) was read on a plate reader (Infinite M200Pro, Tecan, Switzerland). Signal to noise (S/N) was higher at 580 nm than at the 530 nm emission maximum. Concentration was calculated as mg/mL and multiplied by total urine volume in mL, giving total mg in urine. The mg in urine/mg ingested × 100 yielded % ingested.

#### 2.5.3. C18 solid phase extraction of samples for lactulose and mannitol

To remove riboflavin and other colored components, 2 mL urine was processed twice through solid phase extraction (SPE) using 500 mg C18 columns fitted onto a Waters/Millipore SPE vacuum manifold (max −50 kPa, ∼0.5 mL/min). The SPE column was cleaned with MeOH and H_2_O between runs. Mannitol and lactulose recovery were both ∼91%. Data were corrected for a 9% loss. After SPE, samples were directed to the various assays.

#### 2.5.4. Viologen method for lactulose and mannitol permeability in humans

Ready-made assay 96 well plates were prepared (#3694, Corning, USA). A 4× premix buffer was prepared (0.1 M sodium phosphate, 0.1 M HEPES, 0.04% Triton X-100, pH 7.4). To this was added HPTS (16 μM) and quencher (1.6 mM 4,4′oBBV or 2.0 mM 4,4′oMBV), each 4 times above final concentration. The different viologen concentrations were chosen to achieve similar extents of quenching in the absence of sugar (∼20% of free fluorophore) while preserving S/N. Blank wells were given 10 μL 4× premix buffer with neither HPTS nor viologen. Some wells received 16 μM HPTS without quencher to determine maximum possible fluorescence. All other wells received 10 μL of the complete 4× premix. Those premixes containing 4,4′oBBV were continuously vortexed because the mixture is a suspension. Plates were sealed with plate tape and stored at 4 °C until use.

Upon running an assay, 30 μL of mannitol or lactulose standards or samples were pipetted into wells. To explore selectivity, concentration dependencies were determined for the following analytes: sucrose, sucralose, turanose, galactose, fructose, glucose and lactose, each prepared and run in same way. Final reagent concentrations were 4 μM HPTS and 400 μM 4,4′oBBV or 500 μM 4,4′oMBV. Urine samples were placed in both blank wells (for individual sample blanking) and wells containing complete 4× premix. Plates were put on a shaker for 1 hr, RT. During this time, sugars interacted with the HPTS-viologen complex, liberating HPTS into solution. Plates were then centrifuged at 2500 RCF, 10 min, RT to pull down remaining HPTS-quencher particulate matter; plate tape was removed and fluorescence read on a plate reader (Tecan M-200 Infinite, gain 70, 404/535 nm). The height was adjusted to read from the top of the solution (18 mm). This wavelength combination is pH insensitive and poorly affected by any residual riboflavin or endogenous fluorophores that might still be present after C18 SPE. A Marquardt 4-parameter curve fit was used. Sugar concentrations were calculated as g/mL and multiplied by total urine volume in mL, giving total g in urine. Values were corrected for % recovery from the C18 SPE step. The g in urine/g ingested × 100 yielded % ingested. Lactulose measurements were confirmed by enzyme assay [14]. Mannitol measurements were confirmed by HPLC-ELSD using a C8 pre-column and Prevail Carbohydrate ES 5 μ 250 × 4.6 mm column (Grace Davison Discovery Sciences, IL, USA) with 80:20 MeCN/H_2_O.

#### 2.5.5. Statistics

The lower limit of detection (LLOD) was defined as the analyte concentration in the urine sample at which fluorescence intensity in the assay was 3 standard deviations above the mean baseline fluorescence. Similarly, the lower limit of quantification (LLOQ) was defined as 10 standard deviations above the mean baseline fluorescence. CV% was determined by averaging sets of samples each measured in 4 wells. Results are given as mean ± SEM.

## 3. Results

### 3.1. Concentration dependencies and selectivity

**Fig. 2:**
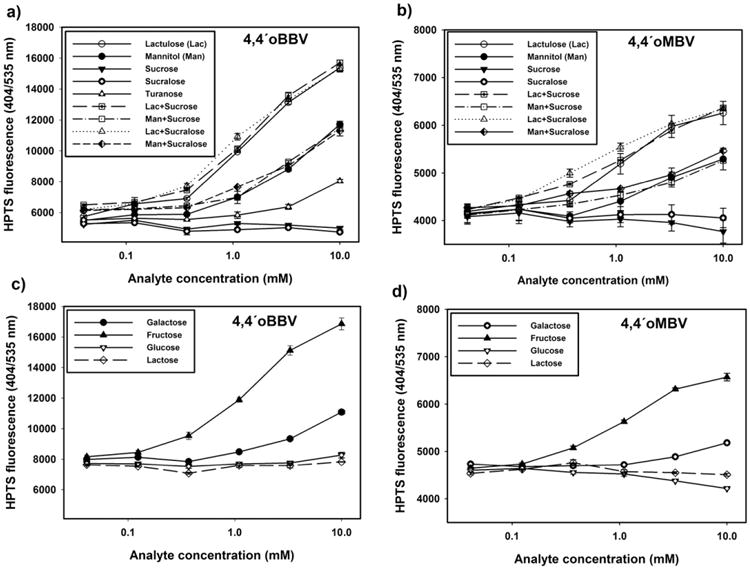
Concentration dependency of the viologen based assay with human urine. (a) Standard curves for lactulose and mannitol using 4,4′oBBV demonstrating strongest sensitivity to lactulose and strong discrimination against sucrose and sucralose. Note the response to the sucrose analog turanose in which C3 of the fructose moiety is occupied by the glycosylic linkage to glucose and cannot take part in binding to boronic acid. (b) Corresponding standard curves for 4,4′oMBV showing similar pattern of responses albeit with weaker changes in fluorescence. (c) Auxiliary data showing comparatively stronger sensitivity of fructose over galactose, the two moieties of lactulose. Note the lack of sensitivity of this assay system for glucose and lactose, which can appear in some patient urine samples. (d) Corresponding data for 4,4′oMBV. Data points are mean ± SEM, n = 3. Concentrations are threefold serial dilutions from 10 mM. Lowest data points are without addition of analytes.

Fig. 2 shows standard curves used to determine limits of detection and quantification for lactulose and mannitol as well as comparisons to other sugars. Limits of detection and quantification are tabulated in Table 1. Also shown are concentration ranges and intra-assay CV%. To illustrate the sensitivity of the assay relative to values obtained in urine samples, the range of concentrations are given instead of % ingested normally stated in a clinical report. The riboflavin assay was at least two orders of magnitude more sensitive than required using the current 50 mg dose. Because this assay measures intrinsic riboflavin fluorescence, standards were linear. For the viologen based sugar assays, the initial quench (as percent of maximum HPTS fluorescence in absence of viologen) indicates that HPTS fluorescence is strongly quenched at the lowest (0 μM) sugar concentration of the standard curve. This also illustrates that 4,4′oMBV is a less potent quencher than 4,4′oBBV, requiring about 20% higher concentration to achieve the same extent of quenching. As sugar concentration increased, 4,4′oBBV showed stronger de-quenching than 4,4′oMBV, indicating 4,4′oBBV has superior ability to resolve different sugar concentrations. Gut permeability assays therefore used 4,4′oBBV. Given that lactulose absorption is very low in healthy subjects, the lower LLOQ of 4,4′oBBV also made it better suited for the gut permeability test. The low CV% achieved (intra-assay) throughout is afforded by the limited number of pipetting steps.

### 3.2. Effectiveness of C18 cleanup

**Fig. 3:**
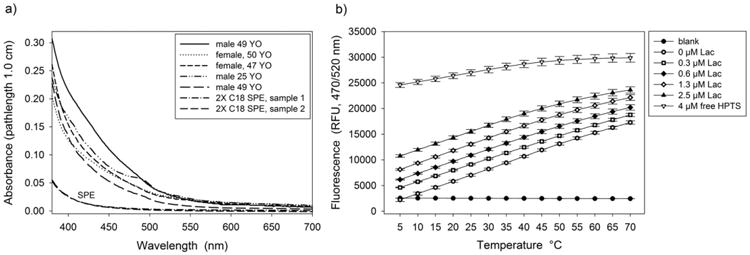
a) Baseline absorbance spectra from 5 human volunteers prior to ingesting permeability probes. Baseline urine samples were used in order to highlight removal of endogenous chromophores. Due to spectral overlap, the result of solid phase extraction (SPE) is only shown for the first two subjects, the first having the highest absorbance obtained in this study. b) Temperature dependency of viologen based fluorescence assay. Magnitude of increase in fluorescence remains relatively constant across the temperature range 10-50 °C. Some of the signal increase is an intrinsic property of HPTS, as seen with 4 μM HPTS in the absence of viologen (open triangle with highest signal). In the absence of sugar at or below 5 °C, signal obtained can dip below blank values, presumably due to absorbance in presence of weaker fluorescence. Data points are mean ± SEM, n = 6. Samples are baseline urine samples spiked with lactulose.

Fig. 3a shows the absorbance of a typical range of urine samples as they are received and the effectiveness of C18 solid phase extraction. After 2 extraction cycles, at which stage no further cleanup can be achieved, urine remained opaque below ∼380 nm and negligible above 700 nm. Variation in absorbance between samples is largely removed. This proved adequate for the 404/535 nm wavelengths of HPTS. Background fluorescence was reduced to ∼20 times below the bottom of the standard curves.

### 3.3. Temperature and solubility

Temperature and solubility of 4,4′oBBV were evaluated because boronic acid moieties of organoboranes generally confer reduced solubility on organoboranes. We have noted a precipitate in the presence of HPTS. Solubility increases in the presence of lactulose. Previous studies using homogeneous buffer systems indicated the optimal quencher/dye ratio to be 125:1 with 500 μM 4,4′oBBV. This was among the best quencher-dye pairings for lactulose measurements. It was also known to result in a precipitate that affected assay performance, perhaps due to the planar symmetry of 4,4′oBBV. We therefore examined the properties of the 4,4′oBBV-HPTS precipitate. It proved to be inversely proportional to sugar concentration and HPTS fluorescence. Following centrifugation, ∼90% of HPTS and a similar amount of 4,4′oBBV was trapped in the pellet. When used to assay sugars, the supernatant of the 4× premix performed poorly compared to the re-suspended precipitate due to loss of most of the fluorophore and viologen. The thermal dependency of the combination of 500 μM 4,4′oBBV and 4 μM HPTS (final concentrations) for lactulose standards in urine is illustrated in Fig. 3b. When temperature and sugar concentration are low, HPTS fluorescence approaches that of the blank. The absorbance contributed by 4,4′oBBV and quenched HPTS in the absence of fluorescence leads to values below the blank, yielding negative values after blank subtraction. A final concentration of 4,4′oBBV (400 μM) was therefore chosen for assays, giving higher S/N at low sugar concentrations. Establishing that the precipitate is a component of the assay led to the practice of centrifuging the plate prior to reading (2500 RCF, 10 min). The plate reader was then configured to read from the top of the solution, thus removing any influence of residual unreacted precipitate. Separate experiments confirmed that this is relevant for the pH insensitive 404/535 nm wavelengths used in an actual permeability test.

### 3.4. Temporal overlap of mannitol and riboflavin

**Fig. 4:**
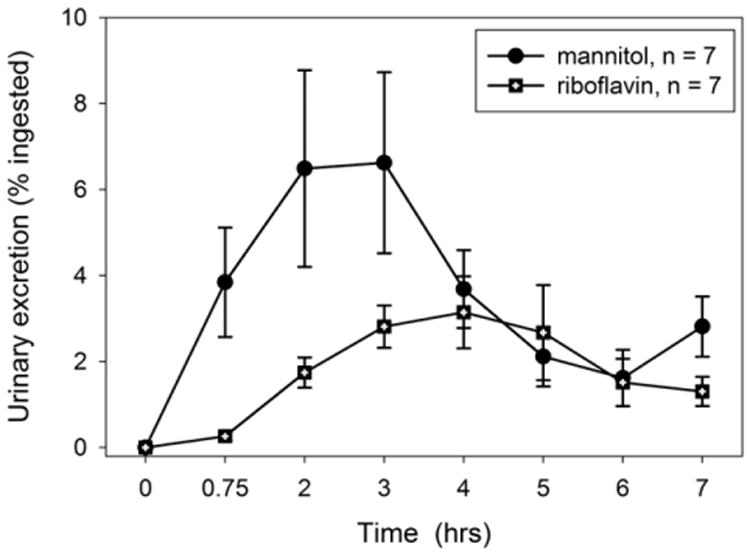
Temporal measurement of mannitol and riboflavin in healthy human volunteers. Mannitol was measured using 4,4′oBBV and riboflavin by autofluorescence (450/580 nm). All data points are mean ± SEM. Riboflavin recovery over 6 hours was 11.1 ± 1.9%.

Fig. 4 shows temporal appearance of riboflavin and mannitol in urine when sampled hourly after ingestion. Riboflavin consistently appeared somewhat later than mannitol. Both riboflavin and mannitol returned to baseline at about 6 hrs, demonstrating that 6 hrs is an acceptable cutoff for studies of small intestinal permeability. Thus, riboflavin, a true nutrient absorbed by way of transport through RFT2, should be able to replace mannitol, which is used as a surrogate marker for nutrient malabsorption, as in active celiac disease.

### 3.5. Lactulose and the lactulose/riboflavin ratio

**Fig. 5:**
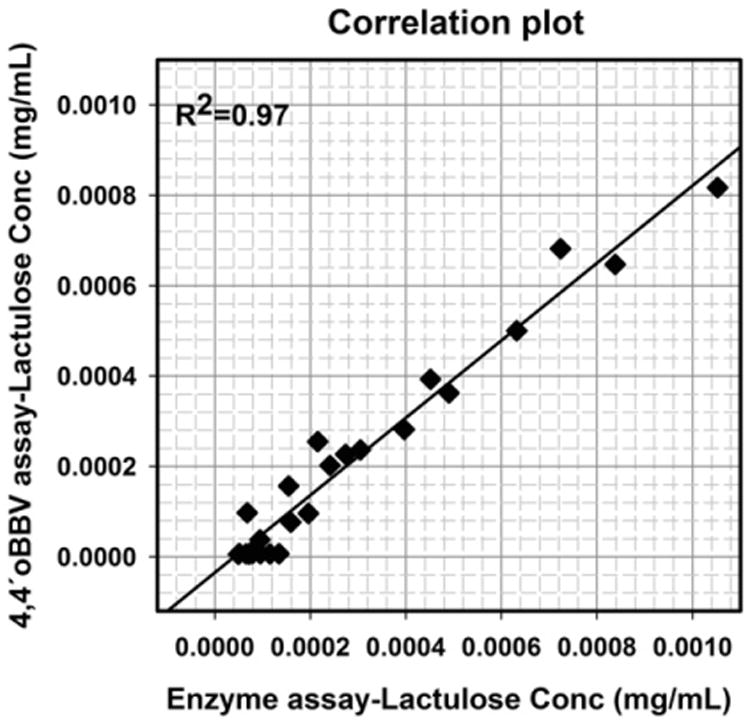
Correlation between the novel 4,4′oBBV fluorescence lactulose assay and conventional enzyme assay. Standards and 24 urine samples were processed through both assays. The novel 4,4′oBBV based assay was run in quadruplicates to ensure accurate measurement of sample CVs, which was 12% for this dataset. The enzyme assay was run in duplicates with a CV of 8%.

Table 2 lists results for lactulose measured in 13 human volunteers using 4,4′oBBV and riboflavin for the time interval 0–6 hrs. Three of ten healthy un-medicated volunteers also performed the test without ingesting lactulose or riboflavin to establish a baseline (B1-B3). Three volunteers had been taking NSAIDs for chronic pain; two of these were outliers in the assays, presumably due to NSAID use. The correlation plot shown in Fig. 5 illustrates the ability of the 4,4′oBBV based fluorescence assay to quantify urine lactulose with results comparable to the established enzymatic assay (n = 24 urine samples).

## 4. Discussion

It was hoped 4,4′oMBV would perform as well as 4,4′oBBV because it is ∼20 times more soluble. In a separate series of experiments reproducing the results of Fig. 2, it was found that a 4,4′-viologen lacking boronic acids wasaneven weaker quencher. This means that boronic acids facilitate HPTS quenching and that facilitation is lost when sugars react with boronic acid groups. Two boronic acids provide more facilitation to quenching, and as a result, more capacity to de-quench in the presence of sugar.

At alkaline pH, boronic acids act by reversible formation of cyclic esters with selective diols within saccharides. We devised two viologens demonstrating high sensitivity and selectivity for lactulose. The binding mechanism of boronic acids to diols has been investigated for glucose and fructose [23,24]. ^13^C-NMR demonstrated that phenylboronic acid forms the β-D-fructofuranose complex or β-D-fructopyranose at C2 and C3 under alkaline conditions similar to this study. Since lactulose contains a fructose moiety in which hydroxyls at C2 and C3 are available, the present viologens were anticipated to interact at these carbons, with galactose potentially contributing a smaller component. Unexpectedly, turanose, a glucose-(1 → 3)-fructose analog of sucrose differing only in the glycosylic linkage at C3 of fructose, gave ∼20% of the lactulose fluorescence, and ∼30% of fructose, whereas sucrose gave no signal. Hence, C3 of the fructose moiety likely accounts for most of the boronic acid binding to lactulose in this assay system, but is not absolutely required. Lactulose gave ∼20% stronger signal than fructose while equimolar amounts of fructose with galactose gave a higher signal than lactulose. Relative selectivity of 4,4′oBBV was lactulose > fructose > galactose, whereas 4,4′oMBV was similar for lactulose and fructose. We therefore propose that lactulose binds 4,4′oBBV in a bidentate (cooperative) fashion involving both the fructose and galactose moieties, explaining the selectivity and sensitivity of this assay for lactulose.

Among the concerns in performing any light based assay on urine is interfering absorbance and fluorescence. For samples destined for the viologen assay, the C18 SPE cleanup procedure proved sufficient to eliminate riboflavin as well as endogenous fluorescence to the extent of being negligible. When high throughput is desired, 96 or 384 well filter plates loaded with C18 may be used with a plate centrifuge.

The upward trend in signal intensity was parallel across all sugar concentrations with rising temperature. This trend was seen with other sugars and with 4,4′oMBV (data not shown). Hence, nothing is achieved by straying from RT, albeit temperature must be stable. The problem of limited solubility of 4,4′oBBV-HPTS was solved by centrifuging the plate and reading fluorescence from the top of the solution.

Sucrose and sucralose are often used in permeability tests to assess gastroduodenal and colonic permeability. Since neither sucrose nor sucralose interfere with the viologen based method, there are no obstacles to their inclusion in clinical studies. Note also that 4,4′oBBV gave essentially no response to 10 mM glucose or lactose. Their presence in urine does not complicate this assay. The mere presence of cis-diols is not sufficient to generate a signal.

Riboflavin, reflecting transcellular absorptive capacity of villi, can replace mannitol. Despite anticipated concerns for variation due to metabolism, etc., riboflavin values in healthy subjects varied, if anything, less than mannitol. The delay in appearance of riboflavin relative mannitol might reflect differences in where the initial uptake occurs. Riboflavin is confined to uptake through the RFT2 transporter and may more strongly correlate with condition of villi tips of duodenum and jejunum. Because RFT2 transport is down-regulated in some gastrointestinal diseases, riboflavin measurements should serve to identify such conditions. The range in riboflavin values obtained here suggests a narrow range of values in healthy individuals.

Regarding predictive value, the three volunteers taking NSAIDs in Table 2 varied in different ways. Subject 11, who had been taking two different NSAIDs during several years for chronic pain, had the highest lactulose recovery as well as lowest riboflavin recoveries thus far. Subject 12 was an asymptomatic type 2 diabetic taking occasional low doses of NSAIDs with recovery values comparable to healthy controls. Subject 13 had the second highest lactulose recovery seen to date, while riboflavin absorption could not have been impaired. Subjects 11 and 13 were originally recruited as healthy volunteers. Their use of NSAIDs was discovered upon queering after the data were obtained. These cases indicate the capacity to identify increased small intestinal permeability. Subject 11 further suggests detection of villi tip architectural damage.

## 5. Conclusions

Reagent costs for the viologen method together with riboflavin were negligible, <1 USD/sample. Reagent costs for enzyme assays (lactulose and mannitol) are ∼25 USD/sample, while requiring more time. The viologen assay permits routine monitoring, which has not been feasible previously. Viologens have remained stable for >4 years. In conclusion, organoboronic acids show potential to study intestinal permeability, in this case together with riboflavin. Future developments of organoborane chemistry for this application are anticipated.

## Figures and Tables

**Table 1:** Lower limits of detection (LLOD) and quantification (LLOQ), range and CV% for riboflavin, lactulose and mannitol in human urine. Estimated from 3 separate runs (3 different occasions) in which all parameters were assayed in parallel. All data points were measured in duplicate on all occasions. Background (assay noise level) and standard deviation for organoboranes were defined as baseline urine with HPTS (404/535 nm) and viologen in the absence of sugar. This 0 μM sugar standard is the point at which maximum HPTS quench is achieved. Values for 4,4′oBBV are averages for 2 different batches.

Parameter	Intl. Quench		LLOD	LLOQ	RANGE	CV%
			
		%max	μM	μM	μM	
Riboflavin						
		N/A	<0.10	0.30	29.3 ± 6.8	<5
Lactulose	4,4′oBBV	24	90	364	132 ±59	3.3
	4,4′oMBV	16	108	704		
Mannitol						
	4,4′oBBV	24	416	860	3929 ± 972	2.4
	4,4′oMBV	16	354	1250		

Initial quench: HPTS fluorescence in presence of quencher and absence of sugar (maximum achievable quench) as a percent of maximum possible fluorescence with 4 μM free HPTS in absence of any quencher. LLOD: analyte concentration in original urine sample (as opposed to final concentration in the assay) at which the fluorescence signal equals or exceeds 3 standard deviations above mean assay noise level. LLOQ: analyte concentration in original urine sample at which the fluorescence signal equals or exceeds 10 standard deviations above mean assay noise level. Range: urine concentrations found in healthy volunteers before transforming data to % ingested. Mean ± SEM. CV%: intra-assay coefficient of variation for samples.

**Table 2:** Lactulose/Riboflavin ratios for healthy (asymptomatic) adult human volunteers. Participants declared no gastrointestinal disturbances and had not been taking NSAIDs or alcoholic beverages immediately prior to or during test (n = 10). Three of these volunteers performed a second test, ingesting water without lactulose or riboflavin to establish a baseline (B1–B3). Doses were 10 g lactulose and 50 mg riboflavin. Urine was collected in a single container for 6 hrs following ingestion, representing small intestinal absorption. For comparison, 3 volunteers who had been taking NSAIDs for chronic pain (otherwise healthy) are listed separately.

Sample	Age	Gender	Lactulose	Riboflavin	Lactulose/Riboflavin
		
			% Ingested	% Ingested	Ratio
B1	33	Female	ND	ND	-
B2	49	Male	ND	ND	-
B3	50	Male	ND	ND	-
——					
1	50	Male	0.268	16.931	0.016
2	50	Male	0.212	9.850	0.022
3	49	Male	0.227	9.300	0.025
4	28	Female	0.149	9.383	0.016
5	41	Female	0.175	6.406	0.027
6	28	Female	0.216	9.469	0.023
7	33	Female	0.142	9.467	0.015
8	20	Male	0.771	17.150	0.045
9	45	Female	0.155	16.136	0.010
10	31	Male	0.501	13.356	0.037
Mean	38		0.282	11.745	0.024
SEM	3		0.064	1.211	0.003
*Volunteers taking NSAIDs*					
11	48	Male	2.240	2.930	0.765
12	59	Male	0.319	10.810	0.030
13	60	Female	0.929	16.701	0.056

ND = not detectable. Signal from raw data was below detection limit and not significantly different from buffer without lactulose or riboflavin.

## Abbreviations

ELSD: evaporative light scatter detector
NSAID: non-steroidal antiinflammatory drug
HPTS: 8-hydroxypyrene-1,3,6-trisulfonate
4: 4′oBBV, 1,1′-bis(2-boronobenzyl)-4,4′-bipyridinium
4: 4′oMBV, 1-benzyl-1′-(2-boronobenzyl)-4,4′-bipyridinium
CV%: coefficient of variation
LLOD: lower limit of detection
LLOQ: lower limit of quantification
RFT2: type 2 riboflavin transporter
S/N: signal to noise ratio
SPE: solid phase extraction
EtOH: ethanol
MeCN: acetonitrile
MeOH: methanol
NAD(P): NAD or NADP
NAD(P)H: NADH or NADPH

## References

[1] Vaarala O (2012). Is the origin of type 1 diabetes in the gut?. Immunol Cell Biol.

[2] Everard A, Cani PD (2013). Diabetes, obesity and gut microbiota. Best Pract Res Clin Gastroenterol.

[3] Pastorelli L, De Salvo C, Mercado JR, Vecchi M, Pizarro TT (2013). Central role of the gut epithelial barrier in the pathogenesis of chronic intestinal inflammation: lessons learned from animal models and human genetics. Front Immunol.

[4] Camilleri M, Madsen K, Spiller R, Greenwood-Van Meerveld B, Verne GN (2012). Intestinal barrier function in health and gastrointestinal disease. Neurogastroenterol Motil.

[5] Piche T (2014). Tight junctions and IBS - the link between epithelial permeability, low-grade inflammation, and symptom generation?. Neurogastroenterol Motil.

[6] Forsyth CB, Shannon KM, Kordower JH (2011). Increased intestinal permeability correlates with sigmoid mucosa alpha-synuclein staining and endotoxin exposure markers in early Parkinson's disease. PLoS ONE.

[7] Clark WF, Sontrop JM, Macnab JJ (2010). Long term risk for hypertension, renal impairment, and cardiovascular disease after gastroenteritis from drinking water contaminated with Escherichia coli O157:H7: a prospective cohort study. BMJ.

[8] Fasano A (2011). Zonulin and its regulation of intestinal barrier function: the biological door to inflammation, autoimmunity, and cancer. Physiol Rev.

[9] Johnson RJ, Rivard C, Lanaspa MA (2013). Fructokinase, Fructans, intestinal permeability, and metabolic syndrome: an equine connection?. J Equine Vet Sci.

[10] Yáñez JA, Teng XW, Roupe KA, Fariss MW, Davies NM (2013). Chemotherapy induced gastrointestinal toxicity in rats: involvement of mitochondrial DNA, gastrointestinal permeability and cyclooxygenase-2. J Pharm Pharm Sci.

[11] Webb DL, Rudholm-Feldreich T, Gillberg L (2013). The type 2 CCK/gastrin receptor antagonist YF476 acutely prevents NSAID induced gastric ulceration while increasing iNOS expression. Naunyn Schmiedeberg's Arch Pharmacol.

[12] Elia M, Behrens R, Northrop C, Wraight P, Neale G (1987). Evaluation of mannitol, lactulose and 51Cr-labelled ethylenediaminetetra-acetate as markers of intestinal permeability in man. Clin Sci (Lond).

[13] van Wijck K, van Eijk HM, Buurman WA, Dejong CH, Lenaerts K (2011). Novel analytical approach to a multi-sugar whole gut permeability assay. J Chromatogr B Analyt Technol Biomed Life Sci.

[14] Northrop CA, Lunn PG, Behrens RH (1990). Automated enzymatic assays for the determination of intestinal permeability probes in urine. 1. Lactulose and lactose Clin Chim Acta.

[15] Lunn PG, Northrop CA, Northrop AJ (1989). Automated enzymatic assays for the determination of intestinal permeability probes in urine. 2. Mannitol Clin Chim Acta.

[16] Subramanian VS, Subramanya SB, Rapp L, Marchant JS, Ma TY, Said HM (1808). Differential expression of human riboflavin transporters -1, -2, and -3 in polarized epithelia: a key role for hRFT-2 in intestinal riboflavin uptake. Biochim Biophys Acta.

[17] Eli M, Li DS, Zhang WW (2012). Decreased blood riboflavin levels are correlated with defective expression of RFT2 gene in gastric cancer. World J Gastroenterol.

[18] Huang S, Jia M, Xie Y, Wang J, Xu W, Fang H (2012). The progress of selective fluorescent chemosensors by boronic acid. Curr Med Chem.

[19] Cao H, Heagy MD (2004). Fluorescent chemosensors for carbohydrates: a decade's worth of bright spies for saccharides in review. J Fluoresc.

[20] Schiller A, Wessling RA, Singaram B (2007). A fluorescent sensor array for saccharides based on boronic acid appended bipyridinium salts. Angew Chem Int Ed Engl.

[R21] 21National Glycohemoglobin Standardization Program (NGSP), USA. http://www.ngsp.org/docs/methods.pdf: a. Ceragem Medisys Inc. (Cera-Stat 2000), b. Infopia Co, Ltd. (Frontier and HemoCue), c. Quotient Diagnostics Ltd. (Quo-Lab), d. Bio-Rad (Deeside in2it A1C), e. Alere Medical Co. Ltd. (Quo-Lab A1c), f. Human GmbH (fluorescence quenching technology), g. Axis-Shield PoC AS (Afinion, NycoCard), h. Trinity Biotech (TriSTAT).

[22] Camara JN, Suri JT, Cappuccio FE, Wessling RA, Singaram B (2002). Boronic acid substituted viologen based optical sugar sensors: modulated quenching with viologen as a method for monosaccharide detection. Tetrahedron Lett.

[23] Norrild JC, Eggert H (1995). Evidence for mono- and bisdentate boronate complexes of glucose in the furanose form Application of 1JC-C coupling constants as a structural probe. J Am Chem Soc.

[24] Norrild JC, Eggert H, Norrild JC, Eggert H (1996). Boronic acids as fructose sensors Structure determination of the complexes involved using (1)J(CC) coupling constants. J Chem Soc Perkin Trans.

